# Adherent but Not Suspension-Cultured Embryoid Bodies Develop into Laminated Retinal Organoids

**DOI:** 10.3390/jdb9030038

**Published:** 2021-09-10

**Authors:** Bojana Radojevic, Shannon M. Conley, Lea D. Bennett

**Affiliations:** 1 Department of Ophthalmology, University of Oklahoma Health Sciences Center, Oklahoma City, OK 73114, USA; Bojana-Radojevic@ouhsc.edu; 2 Department of Cell Biology, Oklahoma Center for Geroscience and Healthy Brain Aging, University of Oklahoma Health Sciences Center, Oklahoma City, OK 73104, USA; Shannon-Conley@ouhsc.edu

**Keywords:** human retinal organoid, retinogenesis, differentiation

## Abstract

Human induced pluripotent stem cells (iPSCs) are differentiated into three-dimensional (3D) retinal organoids to study retinogenesis and diseases that would otherwise be impossible. The complexity and low yield in current protocols remain a technical challenge, particularly for inexperienced personnel. Differentiation protocols require labor-intensive and time-consuming dissection of optic vesicles (OVs). Here we compare this method with a suspension method of developing retinal organoids. iPSCs were differentiated with standard protocols but the suspension-grown method omitted the re-plating of embryoid bodies and dissection of OVs. All other media and treatments were identical between developmental methods. Developmental maturation was evaluated with RT-qPCR and immunocytochemistry. Dissection- and suspension-derived retinal organoids displayed temporal biogenesis of retinal cell types. Differences in retinal organoids generated by the two methods of differentiation included temporal developmental and the organization of neural retina layers. Retinal organoids grown in suspension showed delayed development and disorganized retinal layers compared to the dissected retinal organoids. We found that omitting the re-plating of EBs to form OVs resulted in numerous OVs that were easy to identify and matured along a retinal lineage. While more efficient, the suspension method led to retinal organoids with disorganized retinal layers compared to those obtained using conventional dissection protocols.

## 1. Introduction

Modeling embryogenesis has been an area of scientific research since as early as 1900 with seminal experiments showing reconstruction of autonomously organized, three-dimensional (3D) tissue-like structures from dispersed cell populations (see [1] for more discussion). Current understanding of the formation of laminated vs. non-laminated retinal tissues has been acquired using avian and mammalian embryonic tissue [2,3,4,5,6,7,8]. Tissue engineering based on these concepts has been more recently revolutionized by the innovative knowledge to produce induced pluripotent stem cells (iPSCs) from adult tissues [9,10] enabling ex vivo study of human tissue development. These iPSCs from living human donors can be differentiated into 3D retinal organoids. Differentiation protocols have been improved so that the retinal organoids contain photoreceptors with rudimentary outer segment-like structures and light responses, suggesting functional maturity in culture [11,12,13,14]. Similarities in developmental milestones and expression profiles between the fetal retina and iPSC-derived retinal organoids make organoids a significant and relevant in vitro model for in-depth studies of human retinogenesis that would otherwise be impossible [15].

Retinal degenerative diseases, such as retinitis pigmentosa and other inherited retinal diseases (IRDs), are genetically heterogeneous conditions that lead to severe vision loss and blindness [16]. IRDs are characterized by a high degree of variability and multiple distinct disease mechanisms. In spite of many years of clinical evaluation in patients [17,18,19] and the study of IRD animal models [20,21,22,23], the genetic and phenotypic complexity of IRDs has made a full understanding of disease mechanisms elusive. Even with successful modeling of IRDs using patient-derived iPSCs [24,25,26,27,28], the high degree of complexity and relatively low yield in current protocols remain significant technical challenges, particularly for inexperienced personnel. Current differentiation protocols require labor-intensive and time-consuming dissection of optic vesicles (OVs) [11,29,30] using adherent methods. These complex methods prevent production of retinal organoids on a large scale. Furthermore, the efficiency of the dissection step varies greatly depending on the technical skills of laboratory personnel and their ability to identify OVs, which are sometimes difficult to discern in adherent cultures. Additional variability can also be introduced during plating of the embryoid bodies (EBs), as variable plating density contributes to differences in the effective concentrations of supplements such as Bone Morphogenetic Protein (BMP4). Here we show a modification of the conventional methods for generating retinal organoids from iPSCs, which is simple, time- and resource-saving. We omit the re-plating of EBs to form OVs and simply culture the EBs in a free-floating condition. The resulting OVs were numerous, easy to identify, and matured quickly. However, while more efficient, the suspension method led to retinal organoids with more disorganized retinal layers than those obtained using conventional dissection protocols.

## 2. Materials and Methods

### 2.1. Differentiation Protocol

**Figure 1:**
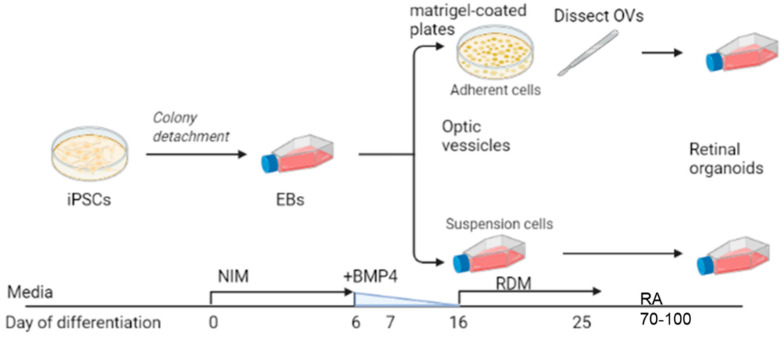
Scheme illustrating dissection vs. suspension differentiation protocols. Embryoid bodies, EBs; Neural induction media, NIM; bone morphogenic protein-4, BMP4; retinal differentiation media, RDM; retinoic acid, RA; optic vesicles, OVs. Image created with Biorender.

We used the iPSC line GM23720 (Coriell Institute, Camden, NJ, USA). Pluripotency was confirmed with immunostaining of multiple pluripotency markers SSEA4, OCT4, TRA-1-60, and NANOG (pluripotency was confirmed by Coriell Institute). The iPSCs were maintained on Matrigel (Corning, NY, USA)-coated plates using StemFlex (ThermoFisher Scientific, Waltham, MA, USA) media. iPSCs were passaged at 60% to 80% confluency using ReLesR (STEMCELL Technologies, Seattle, WA, USA). Differentiation experiments were initiated using an established protocol [31,32]. Figure 1 depicts a brief summary showing iPSC transition into neural induction medium [NIM; DMEM:F12 (1:1; ThermoFisher Scientific), 1% N2 supplement (ThermoFisher Scientific), 1× MEM nonessential amino acids (MEM NEAA; Sigma, St. Louis, MO, USA), 1× GlutaMAX (ThermoFisher Scientific) and 2 mg/mL heparin (Sigma)] for 5 days to promote EB formation. On day 6 (D6), 1.5 nM BMP4 (R&D Systems, Minneapolis, MN, USA) was added to fresh NIM. On D7, EBs were plated on Matrigel (for traditional dissection-based protocols) or transferred to 100-mM polyHEMA (Sigma)-coated flasks (TPP Tissue Culture Flasks; Midwest Scientific, Valley Park, MO, USA) for the suspension protocol. For suspension cultures, there were approximately 100 EBs in the T75 flask. No further manipulation of the suspension culture was performed (such as shaking or rotation). For the dissection cultures, there were ~100 EBs distributed evenly in a 6-well plate. Half of the media was replaced with fresh NIM on D9, D12, and D15. On D16, the media was changed to retinal differentiation medium (RDM; DMEM:F12 3:1, 2% B27 supplement, MEM NEAA, 1× antibiotic, anti-mycotic (ThermoFisher) and 1× GlutaMAX). The media was changed every 2–3 days until OV-like structures appeared, usually by D25, at which time the plated OVs were dissected and transferred to polyHEMA-coated flasks. For the dissection group, retinal organoids that displayed an outer rim of neural retina were identified morphologically by light microscopy and dissected with a MSP ophthalmic surgical knife (Surgical Specialties Corporation, Wyomissing, PA, USA). Retinal organoids were maintained with twice-weekly feeding of 3D-RDM (DMEM:F12 3:1, 2% B27 supplement, 1× MEM NEAA, 1× antibiotic, anti-mycotic, and 1× GlutaMAX with 5% FBS, 100 μM taurine, 1:1000 chemically defined lipid supplement (11905031, ThermoFisher). All-trans retinoic acid (1 μM; Sigma) was included in the media from D70 to D100.

### 2.2. Immunohistochemistry (IHC)

Retinal organoids were fixed in 4% paraformaldehyde (FD neuroTechnologies, Columbia, MD, USA) at room temperature (RT) with gentle agitation for 35–60 min and washed three times with phosphate buffered saline (PBS). Subsequently, retinal organoids were incubated in 15% sucrose in PBS for 1–2 h, transferred to 30% sucrose, and stored at 4 °C overnight. Retinal organoids were embedded in optimal cutting temperature compound and frozen at −20 °C. Cryostat sections (10 μm) were collected using a Leica cryostat onto Superfrost Plus slides and stored at −20 °C in slide boxes prior to immunostaining. Cryosections were air-dried, washed several times in PBS and incubated in blocking solution (10% normal donkey serum (NDS), 5% bovine serum albumin, 1% fish gelatin and 0.5% Triton X-100) for 1–2 h at RT. Primary antibodies were incubated at 4 °C overnight. See Table S1 for a list of primary antibodies, sources and concentrations. Secondary antibodies were diluted to 1:500 and added to tissues for 30 min in the dark at RT (Alexa Fluor 488, AF546 and AF647; ThermoFisher) and again washed with PBS (3 × 10 min). Samples were incubated in DAPI (1:1000, ThermoFisher Scientific) for 5 min, and then washed with PBS (3 × 10 min). Cover slips were mounted over the glass slides, then dried at RT and stored at 4 °C for microscopic observation. Samples were imaged on an Olympus FV1200 confocal microscope.

### 2.3. Quantitative Real-Time Polymerase Chain Reaction (RT-qPCR)

Gene expression was assessed by RT-qPCR. Retinal organoids were pooled (*n* = 3–4) for each time point and homogenized using a Dounce Tissue Grinder (Sigma-Aldrich, Burlington, MA, USA) and processed using SYBR™ Green Fast Advanced Cells-to-CT™ Kit (Invitrogen, Waltham, MA, USA) to make cDNA. RT-qPCR was performed in triplicate using a CFX96 Real-Time System (Bio-Rad, Hercules, CA, USA). Each primer (Table S2) was used at a final concentration of 1 µM. The reaction parameters were as follows: 50 °C for 2 min, 95 °C for 10 min to denature the cDNA and primers, 40 cycles of 95 °C for 3 s followed by primer specific annealing temperature for 30 s (60 °C), succeeded by a melt curve. A comparative cycle threshold (Ct) [33] method was used to calculate the levels of expression that were normalized to *GAPDH* and *β*-*actin*.

### 2.4. Statistics

The difference between the observed means in samples was calculated using independent sample *t*-test, MedCalc Software Ltd (Belgium). Comparison of means calculator. https://www.medcalc.org/calc/comparison_of_means.php (Version 20.009; accessed on 1 July 2021). A value (*p*-value) < 0.05 was considered significant.

## 3. Results

**Figure 2:**
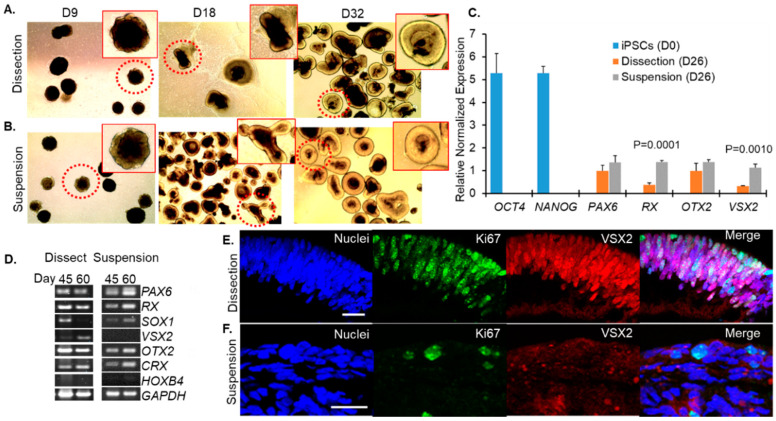
Optic vesicles were identified for (**A**) the dissection and (**B**) suspension protocols. iPSCs lose pluripotency and express eye field transcription factors by day 26 of differentiation. (**C**) RT-qPCR normalized to *GAPDH* and *β*-*actin* on iPSCs and D26 optic vesicles developed using dissection or suspension protocols. (**D**) RT-PCR showing expression of neuroretina progenitors at days 45 and 60 for both methods of differentiation. Representative images from ICC for D50 retinal organoids that were fold homeobox (*RX*) and paired box-6 (PAX6) was detected by D26 in both dissection and suspension groups but not D0 iPSCs, indicating specification of the eye-field (Figure 2C). The earliest specific indicator of neural retina progenitor cells is expression of the gene visual system homeobox 2 (VSX2) which was expressed by developing retinal (**E**) dissected or (**F**) grown in suspension. Scale bar 20 μm.

Our goal was to understand whether mature, properly developed retinal organoids could be generated using a simplified suspension-based protocol rather than the technically challenging dissection based protocols. Using the GM23720 iPSC line, we cultured retinal organoids using these two different methods (referred to as suspension and dissection) as described in Figure 1. Live organoid cultures were imaged by light microscopy to evaluate the timing and overlap of morphological stages using the suspension and dissection protocols. On D9, shortly after dissection protocol organoids had been plated on Matrigel, imaging showed similar shapes and the formation of optic vesicle-like structures in both the dissection (Figure 2A) and suspension (Figure 2B) groups. By D18 and persisting at D32, outgrowths of neural retina were easily identified on organoids from both groups as translucent ovoids surrounding a darker inner core (Figure 2A,B).

The transition from pluripotent stem cells to eye field and optic cup was evaluated by assessing the expression of key regulators of these stages by RT-qPCR in organoids harvested at D26 compared to original iPSCs (D0) (Figure 2C). The pluripotency markers OCT4 (encoded by POU5F1, also known as OCT3, OCT3/4) and NANOG were expressed in iPSCs at D0 but not in D26 organoids from either the dissection or suspension groups (Figure 2C). Expression of retina and anterior neural organoids using both methods of differentiation at D26 but not in iPSCs (Figure 2C). Additionally, developing organoids expressed orthodenticle homeobox 2 (OTX2) by D26 in both groups (Figure 2C). These results showed that the transition from pluripotency to a presumptive eye field and OV formation was evident regardless of which of the two culture methods was used.

Early development of retinal progenitor cells was further evaluated by RT-PCR at D45 and D60 for each method of differentiation. Eye-field genes (PAX6, *RX*) continued to be robustly expressed in both groups at these time points as does the photoreceptor progenitor marker OTX2 (Figure 2D). Both groups also expressed the photoreceptor-specific transcription factor CRX (cone-rod homeobox) at D45 and D60 (Figure 2D). The non-retinal neuronal marker HOXB4 (hindbrain-specification), was expressed at low levels in samples from both dissection and suspension protocols (Figure 2D) consistent with the organoids maturing largely along a retinal branch.

However, the SOX1 (SRY-Box Transcription Factor 1) transcription factor, which is expressed in activated neural stem cells and is involved in specification of rostral hindbrain, was expressed differently between the two developmental methods. Dissected retinal organoids expressed SOX1 at D45 but not D60 whereas those that were grown in suspension expressed SOX1 at both time points (Figure 2D), suggesting that development of suspension-grown organoids may lag behind those grown with the dissection method. This was supported by immunofluorescence labeling performed at D50, with Ki67 (a marker for proliferating cells, green) and VSX2 (neural retina progenitors). Ki67 (green) was prominently expressed in neural retina progenitors (VSX2, red) in dissected retinal organoids (Figure 2E) but these proteins were rarely detected in retinal organoids developed by the suspension method (Figure 2F). Interestingly, VSX2 expression patterns were also different in dissection vs. suspension grown organoids. At D26, we observe higher expression of VSX2 message in suspension cultures compared to dissection cultures (Figure 2C). However, by D45–60, our immunofluorescence and RT-PCR studies show that VSX2 levels are dropping in suspension cultures while remaining higher in dissection cultures (Figure 2D–F). Combined these data suggest that VSX2 expression is turning both on and off earlier in suspension cultures than dissection cultures. We do not observe this pattern with other developmental regulators such as PAX6 and OTX2.

**Figure 3:**
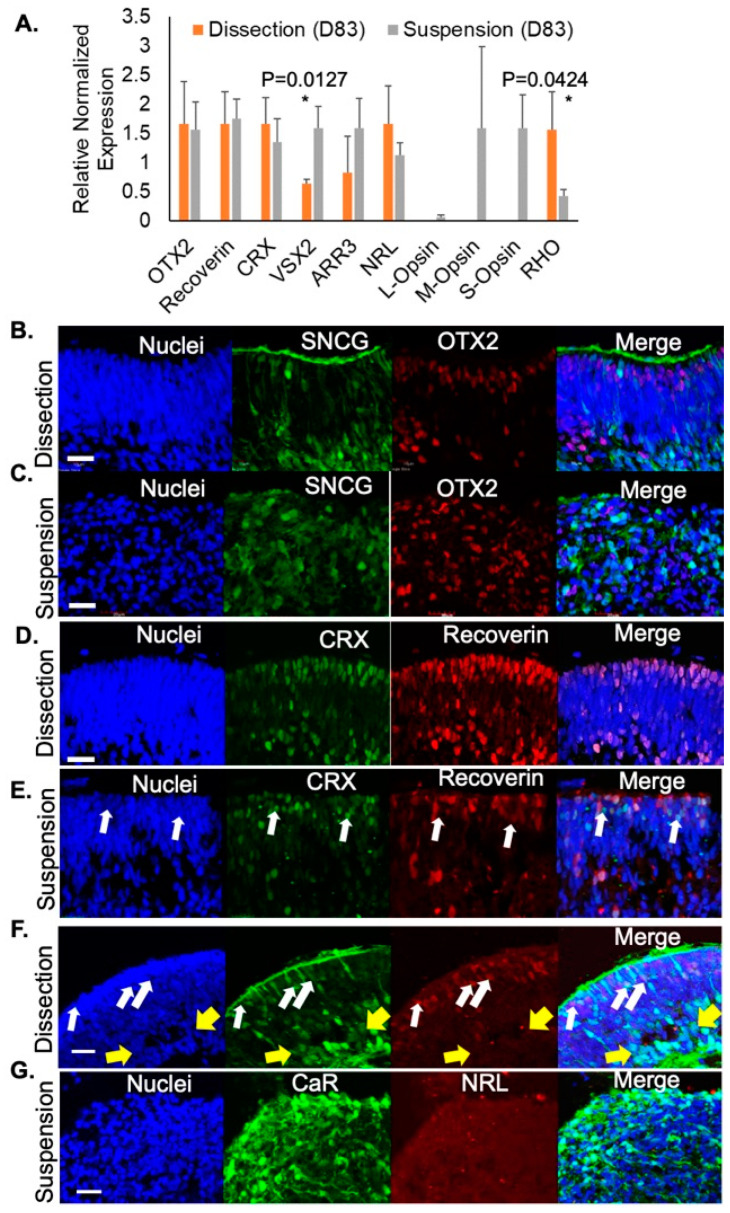
The onset of photoreceptor differentiation was identified in retinal organoids. (**A**) RT-qPCR on D83 retinal organoids showing expression of photoreceptor-specific markers. Representative images showing SNCG (green) and OTX2 (red) expression from D100 retinal organoids grown using the (**B**) dissection (**C**) or suspension method. (**D**–**G**) Representative images from D100 retinal organoids differentiated using the (**D**,**F**) dissection method or the (**E**,**G**) suspension method. White arrows highlight NRL+ rods. Yellow arrow highlights formation of the inner retinal layer. Scale bar 20 μm. * Statistical significance *p* ≤ 0.0500.

Next, we evaluated photoreceptor-specific gene expression in retinal organoids developed using either the dissection or suspension method. At D83, organoids cultured in both protocols exhibited similar levels of expression of photoreceptor precursor genes including OTX2, Recoverin, and CRX (Figure 3A). Interestingly, at this stage, suspension-grown organoids had greater expression of VSX2 (neural retina progenitors) and proteins unique to cone photoreceptors such as cone arrestin (ARR3), long-wavelength opsin (L-Opsin), medium- wavelength opsin (M-Opsin), and short-wavelength opsin (S-Opsin) whereas dissected retinal organoids had greater expression of markers for rod photoreceptors including neural retina leucine zipper (NRL) and rhodopsin (RHO; Figure 3A). These findings suggest that while both suspension-grown and dissected retinal organoids contain developing photoreceptors, rods were enriched in dissected organoids while cones were preferentially found in suspension grown cultures.

Retinal development was further evaluated. From the dissection protocol, retinal organoids exhibited RGCs (SNCG+, green) enriched along the inner aspect of the retinal organoids with photoreceptor cell progenitors (OTX2+, red) in a separate layer in the outer portion of the organoid (Figure 3B). However, this lamination was not present in the organoids from the suspension protocol; though both RGCs and photoreceptor progenitors were detected, they distributed throughout the organoid in a disorganized pattern (Figure 3C). Using immunofluorescence, photoreceptors were identified with co-labeling of CRX (green) and recoverin (red) in D100 retinal organoids (Figure 3D). As expected, in dissection-grown organoids, photoreceptors expressed both CRX and recoverin. In contrast, cells positive for CRX labeling but negative for recoverin (arrows) were found in suspension retinal organoids at D100 (Figure 3E), indicating that these cells have exited the cell cycle but have not yet differentiated fully, a phenotype not seen in dissection protocol organoids at this time point (Figure 3F). This tendency toward delayed photoreceptor maturation and retinal disorganization in suspension grown organoids is also evident by evaluation of NRL (rod marker, red) and CaR (inner retinal neurons) at D100. At this stage, the dissected retinal organoids showed many cells committed to a rod fate (NRL, arrows, red) localized to the outer rim of the organoid and in a separate layer than the RGCs and ACs (CaR, green; Figure 3F). Although some CaR+ cells in dissection grown organoids have not yet fully migrated to their final position in the inner retina, the majority of CaR+ inner retinal cells localize to a distinct inner retinal layer (yellow arrow, Figure 3F). This is in contrast to suspension-developed retinal organoids which continued to be disorganized with RGCs and ACs (CaR) dispersed throughout the tissue and very little NRL (Figure 3G). These results demonstrated the expansion of neural retina progenitor cells within an outer neuroblastic layer using both differentiation methods and a continued disorganized inner retinal layer in organoids grown with the suspension method compared to the laminated inner retinal cells (CaR, RGCs, and ACs; green) of those grown using the dissection method (Figure 3F).

**Figure 4:**
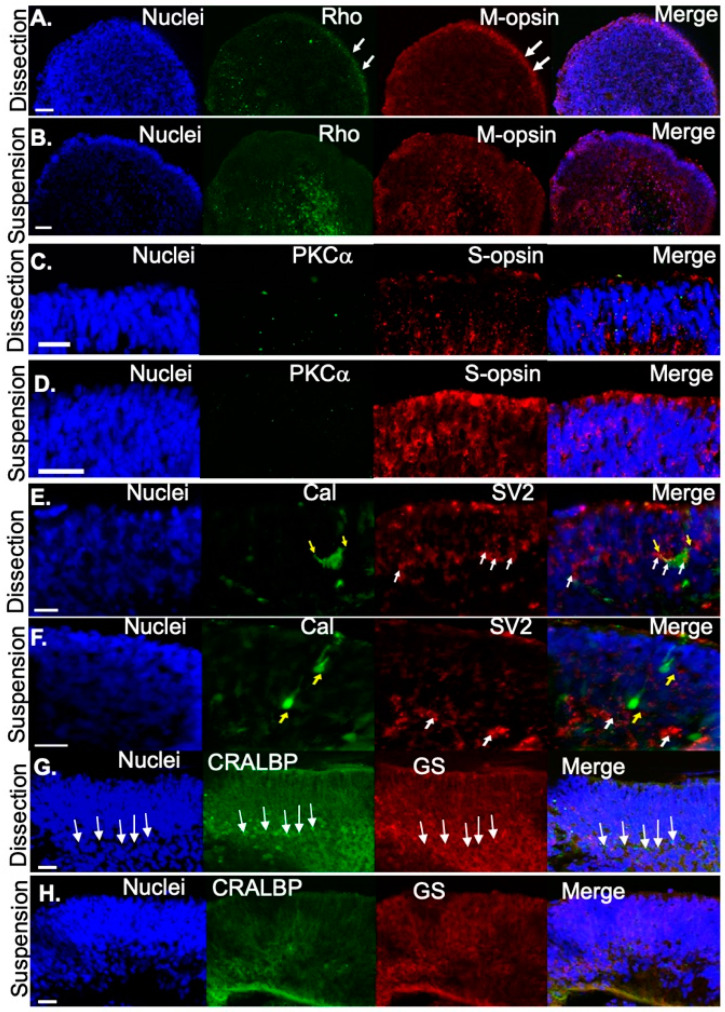
Retinal organoids express photoreceptor proteins at D120. (**A**) Rhodopsin (Rho) and M-opsin were correctly localized to the outer rim in dissected retinal organoids but Rho and M-opsin was centrally or diffusely localized, respectively in (**B**) suspension retinal organoids. S-opsin was rarely detected in (**C**) dissected retinal organoids but was (**D**) localized to the putative outer segments for those grown under suspension. (**E**) Yellow arrows highlight horizontal cells (Calbindin, Cal, green) with processes reaching out to photoreceptor terminals (white arrows, SV2, red). (**F**) Yellow arrows highlight horizontal cells (Cal, green), exhibiting a vertical alignment without connection to the photoreceptor terminals (SV2, red). (**G**) Müller glial cells were identified with CRALBP (arrows) and GS in dissected retinal organoids and to a lesser extent in (**H**) suspension-grown retinal organoids. Scale bars 20 μm.

Subsequently, maturation of photoreceptors was evaluated at D120. Immunofluorescence was performed to compare the distribution of proteins specific to mature photoreceptors in organoids that were dissected versus those that were developed in suspension. Mature rods (Rho, green) and medium-wavelength cones (M-opsin) were found at the outer edge of the presumptive photoreceptor layer in dissected retinal organoids (Figure 4A), but were distributed throughout the organoid in suspension-derived retinal organoids (Figure 4B). In contrast, compared to dissected retinal organoids (Figure 4C), short-wavelength opsin (S-opsin, red) was expressed more prevalently in photoreceptors in suspension-cultured (Figure 4D) retinal organoids. At this stage (D120), neither method of differentiation produced retinal organoids containing mature bipolar cells (PKCα, green; Figure 4C,D). Labeling for the photoreceptor terminal marker SV2 at D120 (red) demonstrated that structures consistent in shape with photoreceptor terminals (white arrows) were detected in both dissection (Figure 4E) and suspension-grown (Figure 4F) cultures. We co-labeled with the calcium binding protein calbindin (green, Figure 4E,F) which is expressed in the human retina in cones, subsets of inner retinal neurons, and some ganglion cells [34,35], and observed calbindin positive cells (yellow arrows, Figure 4E,F) under both culture conditions. However, cells exhibiting horizontal cell morphology, characterized by large squat cell bodies and processes that penetrated horizontally through the tissue (i.e., perpendicular to the length of the photoreceptor) were detected only in dissection grown cultures. In dissection grown cultures, these processes often overlapped with SV2 labeling, suggesting synaptic connections may be forming between horizontal cells and photoreceptors. Although calbindin-positive cells were found in suspension grown cultures (yellow arrows, Figure 4F), they did not exhibit the typical squat cell body morphology, were frequently aligned in parallel with photoreceptors rather than horizontally, and did not exhibit horizontally projecting processes, suggesting they are likely to be other retinal cell types. Immunocytochemical staining at D120 also showed that Müller glia markers including cellular retinaldehyde binding protein (CRALBP; green) and glutamine synthetase (GS; red) were expressed in the retinal organoids. There was more expression in organoids cultured under the dissection protocol compared to the suspension protocol. In addition, in organoids cultured under the dissection protocol, Müller glia nuclei (arrows, Figure 4G) were largely restricted to a defined layer. This organization was absent in cultures grown under suspension conditions. In summary, both methods of differentiation resulted in retinal organoids that contained photoreceptors and some retinal interneurons. However, the dissected retinal organoids displayed better organization of retinal layers than suspension-grown organoids.

## 4. Discussion

In vivo, OVs evaginate from the anterior neural plate which acts as a substrate for the developing retina. In cultured organoids, this step is modeled by the embedding of developing EBs into Matrigel. However, the suspension culture approach we present here circumvents this matrix stage, a step which was previously presumed to be necessary to acquire a neuroectoderm fate. We show that suspension cultures express markers of anterior neuroepithelial commitment (PAX6 and OTX2 [36]) suggesting that the plating of EBs on Matrigel may not be necessary for developing retinal organoids. The Matrigel substrate contains basement membrane proteins such as collagen IV, entactins, and laminins as well as some growth factors. In addition to individual protein components provided by the Matrigel, the physical interaction with the retinal organoids may mimic interactions between extraocular tissues in vivo with the developing retina which has been thought to be vital to development since purified entactins and laminins alone were unable to induce expression of the eye field transcription factor *RX* [37]. However, we were able to detect *RX* when the EBs were not grown on Matrigel at D26, D45, and D60, suggesting that the ECM environment may not be absolutely required for the adoption of retinal cell fates. One difference between our studies and the seminal research by Eiraku et al. [37] evaluating the role of matrix in organoid development is that their starting material was embryonic stem cells isolated from mice whereas our cells were human-derived iPSCs, highlighting the variability in outcomes across species. In addition, the time course of Matrigel culturing was different between the Eiraku study and the protocols we use, so we cannot directly compare *RX* expression between the two studies. However, our findings suggest that culturing on Matrigel is not an essential step for cells to progress along the eye field lineage.

Removing the Matrigel embedding and dissection steps led to a simpler and less labor-intensive protocol that saved time and money compared to traditional dissection-based protocols. Culturing organoids in continual suspension also eliminated the subjective selection of OVs which may not be readily identifiable on adherent cultures due to the similar appearance of non-neural retina organoids. Here, we have shown that suspension-derived retinal organoids displayed temporal development patterns similar to those seen in fetal retinogenesis including biogenesis of early-born retinal cell types, such as RGCs, ACs and HCs, followed by expression of markers for commitment to photoreceptor lineages. However, there were notable differences in the retinal organoids generated by the two methods of differentiation. In addition to some differences in developmental timing, the other striking difference between the two culture methods was in the organization of neural retina layers. The suspension method resulted in retinal organoids that were dis-organized at the layers of the inner and outer retina compared to the dissected retinal organoids. Growth of EBs on Matrigel may contribute to an artificial molecular gradient of factors known to be important for retinogenesis [38,39,40] and thus promote the development of multi-layered, laminated retinal organoids. The absence of these growth cues in suspension grown organoids may contribute to the resulting disorganization of retinal cells. One interesting observation was that VSX2 expression was earlier to turn on and off in suspension vs. dissection grown cultures, and it is not clear why this would be the case. Expression of VSX2 promotes development of bipolar cells at the expense of photoreceptors and elimination of VSX2 suppresses bipolar cell differentiation [41,42], so abnormally early VSX2 expression in suspension cultures may contribute to the overall abnormal formation of the inner retina and its failure to develop into a distinct layer that we observe in suspension cultures. In addition, another key contributor to the lack of lamination in the suspension-cultured retinal organoids may be insufficient Müller glial support [43]. The lamina-promoting role of Müller glia precursors efficiently provide support to achieving organization and their absence in suspension-cultured organoids may be a focus for future studies on the role of Müller glia and the precursor cells in retinal organoids. Additional studies of cultures differentiated by the current suspension protocol may provide insight into regulatory mechanisms that govern migration and final localization of the laminated neural retinal cells in the human retina.

There was variability in the degree of disorganization amongst different suspension-grown specimens. Suspension-grown organoids exhibited some signs of retinal lamination in the outer nuclear layer but there tended to be an overall delay in maturation compared to the dissected retinal organoids. Polarized photoreceptor precursor cells have been observed in retinal organoids as early as D90 [44,45]. Consistent with this, at D100, the photoreceptor cell specific markers recoverin (a protein involved in the photo-transduction cascade expressed by both rods and cones) and CRX were localized to the presumptive photoreceptor layer in retinal organoids developed by either method of differentiation. However, we frequently observed CRX-positive recoverin-negative photoreceptors in suspension grown cultures. These cells in the developing outer nuclear layer represent a subpopulation of photoreceptors that were non-proliferative but had not yet expressed markers (e.g., recoverin) characterizing mature photoreceptors. By D120, we did observe photoreceptors in organoids grown in suspension (as well as dissection) cultures that expressed mature functional photoreceptor proteins such as Rho, S-Opsin, and M-Opsin suggesting that maturation was not completely blocked in suspension cultures.

Overall, the timing of gene expression for markers of retinogenesis in retinal organoid development coincided with that of normal human retinal development with the sequential acquisition of neuroretinal- and photoreceptor-associated gene expression throughout the differentiation process [46]. However, there are well-known intrinsic differences in iPSC derivation, maintenance, storage, and tissue source. Consequently, a caveat to our study is that other iPSC lines may behave differently in dissection vs. suspension culturing due to variations in donors, starting tissue source, and reprogramming protocols that may impact the endogenous expression levels of components of signaling pathways due to epigenetic memory [47,48]. The method described here supports applications requiring large-scale generation of retinal organoids, such as high throughput drug screening when exact retinal lamination is not required. This method could be a potential source of cone-expressing retinal organoids for specific studies of cone photoreceptors. However, the traditional method of dissecting retinal organoids is preferred for studying developmental processes and cellular mechanisms unique to the human retina.

## Data Availability

Access to the images and CT values from RT-qPCR are available upon request.

## Supplementary Materials

The following are available online at https://www.mdpi.com/article/10.3390/jdb9030038/s1, Table S1: Antibodies; Table S2: Primers.
