# Supplementary material for: Adherent but Not Suspension-Cultured Embryoid Bodies Develop into Laminated Retinal Organoids
Source: J Dev Biol. 2021 Sep 10;9(3):38. doi: 10.3390/jdb9030038 (PMC8482155; doi:10.3390/jdb9030038)
Supplement: Supplementary file 1 [file jdb-09-00038-s001.zip › Antibodies.pdf]

Antibodies	detection	type	source	Cat number	Dilution
VSX2 (CHX10)	NRPC, PR precursors; BC	Rabbit polyclonal	ThermoFisher	PA582092	1:200
Calretinin (CaR)	RGC and AC	Rabbit polyclonal	Milipore	AB5054	1:500
Calbindin (Cal)	Horizontal cells (HC)	Chicken polyclonal	Synaptic systems	214006	1:500
CRX	Photoreceptors	Mouse monoclonal	Abnova	H00001406-M02	1:100
Ki67	Proliferation NRPC	Mouse monoclonal	BD Biosciences	556003	1:500
NRL	Early rod	Goat polyclonal	R&D	AF2945	1:300
OC1-ONECUT1	HC progenitor	Sheep polyclonal	R&D	AF6277	1:300
OTX2	PR progenitor	Goat polyclonal	R&D	AF1979	1:2000
pKCα	Bipolar cells	Mouse monoclonal	Thermofisher	MA1-157	1:100
Recoverin	Photoreceptors	Rabbit polyclonal	Chemicon	AB5585	1:2000
SNCG	RGC	Mouse monoclonal	Abnova	H00006623-M01	1:500
RHO	Rods	Mouse monoclonal	Gift from Muayyad Al-Ubaidi	Clone1D4	1:100
SV2	PR synapse marker	Mouse monoclonal	DSHB	AB2315387	1:50
S-Opsin	Blue opsin (cones)	rabbit	Invitrogen	pA5-112636	1:100
L-Opsin	Red opsin (cones)	rabbit	Invitrogen	PA5-98031	1:100
M-Opsin	Green opsin (cones)	rabbit	Invitrogen		1:100
Secondary antibodies: anti-mouse (AF546, AF488, AF647); anti-rabbit (AF488, AF546, AF647); anti-chicken (AF488); anti-sheep (AF546); anti-goat (AF546)					

Neural retinal progenitor cells, NRPC; Development studies hybridoma bank, DSHB
