# Supplementary material for: Adherent but Not Suspension-Cultured Embryoid Bodies Develop into Laminated Retinal Organoids
Source: J Dev Biol. 2021 Sep 10;9(3):38. doi: 10.3390/jdb9030038 (PMC8482155; doi:10.3390/jdb9030038)
Supplement: Supplementary file 1 [file jdb-09-00038-s001.zip › Primers.pdf]

Gene	Forward Primer	Reverse Primer
Oct4 (qPCR)	GTG GAG GAA GCT GAC AAC AA	ATT CTC CAG GTT GCC TCT CA
Nanog	CAA AGG CAA ACA ACC CAC TT	TCT GCT GGA GGC TGA GGT AT
PAX6 (qPCR)	AGT GAA TCA GCT CGG TGG TGT CTT	TGC AGA ATT CGG GAA ATG TCG CAC
RX (qPCR)	AGC GAA ACT GTC AGA GGA GGA ACA	TCA TGC AGC TGG TAC GTG GTG AAA
OTX2 (qPCR)	AGA GCA GCC CTC ACT CGC CA	AGT CGG CCC AAA TCG GGG GT
VSX2 (qPCR)	GGC GAC ACA GGA CAA TCT TTA	TTC CGG CAG CTC CGT TTT C
SOX1	CAA TGC GGG GAG GAG AAG TC	CTC TGG ACC AAA CTG TGG CG
CRX (qPCR)	TGA TGC ACC AGG CTG TGC CCT A	TGG CAA ACA GTG CCT CCA GCT C
HOXB4	GCA AAG AGC CCG TCG TCT AC	CGT GTC AGG TAG CGG TTG TA
GAPDH (qPCR)	TGCACCACCAACTGCTTAGC	GGCATGGACTGTGGTCATGAG
Recoverin	CTC CTT CCA GAC GAT GAA AAC A	GCC AGT GTC CCC TCA ATG AA
ARR3 (qPCR)	AGG AAA GCC CTG TGG GAT TGA CTT	AAC CAG CCG CAC ATA GTC TCT CTT
L-OPSIN (qPCR)	GCCTACTTTGCCAAAAGTGC	GATGAGACCTCCGTTTTGGA
M-OPSIN (qPCR)	CATCTTTGGTTGGAGCAGGTACT	TCTCTGCCTTCTGGGTGGAT
S-OPSIN (qPCR)	ATACCGCAGCGAGTCCTATAC	GATCCTACCATCACAACCAC
NRL (qPCR)	AGT TTG AGG TAA AGC GGG AAC CCT	ACC ATG CCT GGT TCA CTGAAG GT
RHO (qPCR)	TTTGGAGGGCTTCTTTGCCA	CCTCGGGGATGTACCTGGAC
